# Sputum colour as a marker for bacteria in acute exacerbations of COPD: protocol for a systematic review and meta-analysis

**DOI:** 10.1186/s13643-021-01767-6

**Published:** 2021-07-27

**Authors:** Ruan Spies, Matthew Potter, Ruan Hollamby, Stefan van der Walt, Ameer Hohlfeld, Eleanor Ochodo, Richard N. van Zyl-Smit

**Affiliations:** 1 Port Elizabeth Hospital Complex, Port Elizabeth, South Africa; 2 Livingstone Tertiary Hospital, Stanford Road, Port Elizabeth, South Africa; 3 Helen Joseph Hospital, Johannesburg, South Africa; 4 General Justice Gizenga Mpanza Regional Hospital, KwaDukuza, South Africa; 5 Cochrane South Africa, South African Medical Research Council, Cape Town, South Africa; 6 Centre for Evidence-Based Healthcare, Division of Epidemiology and Biostatistics, Faculty of Medicine and Health Sciences, Stellenbosch University, Stellenbosch, South Africa; 7 Centre for Global Health Research, Kenya Medical Research Institute, Nairobi, Kenya; 8 Division of Pulmonology, Department of Medicine, University of Cape Town, Cape Town, South Africa; 9 University of Cape Town Lung Institute, Cape Town, South Africa

**Keywords:** COPD, Acute exacerbation of COPD, Sputum colour, Sputum purulence, Chronic bronchitis, Emphysema

## Abstract

**Background:**

Chronic obstructive pulmonary disease (COPD) is a major cause of years of life lost globally. Acute exacerbations of COPD (AECOPD) drive disease progression, reduce quality of life and are a source of mortality in COPD. Approximately 50% of AECOPD are due to bacterial infections. Diagnosing bacterial infection as the aetiology of AECOPD however remains challenging as investigations are limited by practicality, accuracy and expense. Clinicians have traditionally used sputum colour as a marker of bacterial infection in AECOPD, despite the lack of high-quality evidence for this practice. The aim of this systematic review and meta-analysis is to determine the diagnostic accuracy of sputum colour in the diagnosis of bacterial causes of AECOPD.

**Methods:**

Articles will be searched for in electronic databases (MEDLINE, Google Scholar Scopus, Web of Science, Africa-Wide, CINAHL and Health Source Nursing Academy) and we will conduct a review of citation indexes and the grey literature. Two reviewers will independently conduct study selection, against pre-defined eligibility criteria, data extraction and quality assessment of included articles using the QUADAS-2 tool. We will perform a meta‐analysis using a bivariate logistic regression model with random effects. We will explore heterogeneity through the visual examination of the forest plots of sensitivities and specificities and through the inclusion of possible sources of heterogeneity as covariates in a meta-regression model if sufficient studies are included in the analysis. We also perform a sensitivity analysis to explore the effect of study quality on our findings. The results of this review will be reported according to the Preferred Reporting Items for Systematic Reviews and Meta-analysis statement and will be submitted for peer-review and publication.

**Discussion:**

The findings of this review will assist clinicians in diagnosing the aetiology of AECOPD and may have important implications for decision making in resource-limited settings, as well as for antimicrobial stewardship.

**Systematic review registration:**

PROSPERO CRD42019141498

**Supplementary Information:**

The online version contains supplementary material available at 10.1186/s13643-021-01767-6.

## Background

Chronic obstructive pulmonary disease (COPD) is the 7th leading cause of years of life lost globally [1]. The disease is a major source of chronic morbidity and is associated with a significant economic and social burden [2–4]. Acute exacerbations of COPD (AECOPD) are defined by the acute worsening of respiratory symptoms which require additional therapy [5].

AECOPD increase rates of rehospitalization, drive disease progression, reduce quality of life and are a source of mortality in COPD [6–8]. It is estimated that 50–70% of exacerbations may be due to respiratory infections, including bacteria, atypical bacteria and respiratory viruses [9]. The use of antibiotics in AECOPD is controversial [10, 11]. Current treatment guidelines recommend antibiotic therapy in patients with moderate to severe AECOPD with three cardinal symptoms (increase in dyspnoea, sputum volume and sputum purulence) or two cardinal symptoms including sputum purulence; or in patients who require mechanical ventilation [5]. No definition of purulence is provided, and this assessment is thus left to the clinician’s judgement. A Cochrane review on antibiotic use in AECOPD reported inconsistent treatment effects across different grades of exacerbation severity [12]. Clinicians thus need to carefully consider the benefits of antibiotic therapy in AECOPD against the potential harms, including the emergent public health crisis of antibiotic resistance [13].

Current investigations for the diagnosis of bacterial infections in AECOPD are cumbersome and lack sensitivity [5]. Sputum cultures require at least two days incubation while microbiological analysis is often limited by technical issues related to sample adequacy [5]. In areas remote from laboratory services, access to sputum analysis may not be possible and delays to sample processing and reporting may testing unfeasible. Furthermore, the respiratory tracts of individuals with COPD may be colonized by potentially pathogenic microorganisms, and the detection of bacteria in sputum may not reliably discriminate infection from colonization [14]. Biomarkers also have limited value. C-reactive protein is not specific and although procalcitonin may be more specific for bacterial infections, its use is limited by expense, limited availability and current lack of strong evidence to recommend its use [5, 15]. The analysis of sputum colour is a clinical sign traditionally utilized by clinicians in the assessment of AECOPD. Purulent sputum, typically defined as green, yellow or brown coloured sputum, may result from the increased recruitment of neutrophils into the sputum, with colouring resulting from the green myeloperoxidase present in these cells [16]. This is thought to represent an acute inflammatory response to bacterial infection [16]. The landmark study by Anthonisen demonstrated a significant treatment effect when antibiotics where used in patient with AECOPD and purulent sputum [17]. However, this study did not investigate the sputum bacteriology of the participants and there is subsequently a lack of high-quality evidence supporting the use of sputum colour as a diagnostic marker in AECOPD [12].

Sputum colour analysis may be an attractive option for clinicians, should it prove to be an accurate marker of bacterial infection. It is a rapid assessment that can be made immediately at the bedside, allowing for early initiation of appropriate antibiotic therapy. Furthermore, sputum colour assessment tools have been developed which may help improve the consistency and accuracy of such measurement [18].

There are, to the best of our knowledge, no existing systematic reviews of this subject. It is unlikely that sputum colour alone will be useful in determining the presence of bacterial infection in AECOPD. However, it will benefit clinicians to better understand the sensitivity and specificity of sputum colour assessment so that it may be used appropriately in context with other clinical, laboratory and radiological findings. In resource limited settings, where access to radiology, biomarkers and microbiology may be unavailable, understanding the sensitivity and specificity of sputum colour as a marker for bacteria in AECOPD may improve the accuracy of clinical diagnosis, minimize patient waiting times due to sample transport and reduce subsequent loss to follow up.

### Objectives

The primary objective of this systematic review is to evaluate the diagnostic accuracy of sputum colour as a marker for the presence of bacteria in the sputum of adults with AECOPD.

## Methods

This protocol was registered with the International Prospective Register of Systematic Reviews (PROSPERO) on 27 September 2019 with registration number CRD42019141498 and has been written in accordance with the Preferred Reporting Items for Systematic Review and Meta-analyses Protocols (PRISMA-P) guidelines [19] (See checklist in Additional file 1). The final review will be reported in accordance with the Preferred Reporting Items for Systematic Reviews and Meta-analyses extension for Diagnostic Test Accuracy studies extension (PRISMA-DTA) [20].

### Eligibility criteria

A study will only be deemed eligible for review if it fulfils the inclusion criteria and if it does not fulfil any of the exclusion criteria as demonstrated in Table 1.

**Table 1:** Review eligibility criteria

**Inclusion criteria**	**Exclusion criteria**
***Participants***	
Participants diagnosed with acute exacerbation of COPD	Stable COPD
***Index test***	
Sputum colour macroscopically assessed by professional healthcare worker or patient reported	
***Reference standard***	
Quantitative culture for potentially pathogenic bacteria on an adequate sputum sample (according to the Murray-Washington or Bartlett criteria)	
***Outcome***	
Data required to populate 2 X 2 table: sensitivity and specificity or true positives, false positives, false negatives and true negatives	
***Study design***	
Prospective studies	Case reports
Retrospective studies	Case series
Cross-sectional studies	Ecological studies
	Diagnostic case control studies with healthy controls

#### Types of studies

We will include observational studies (including prospective, retrospective and cross-sectional studies) and randomized-controlled trial studies which report the accuracy of sputum colour in identifying the presence of bacteria in AECOPD. We will not restrict studies by language or publication date. We will only include studies in which the data required to populate 2 X 2 tables are reported, can be reconstructed from reported summary estimates or can be provided by the authors of primary diagnostic studies. The data include true positives, false positives, true negatives and false negatives.

Case reports, case series, diagnostic case–control studies with healthy controls and studies presenting insufficient data for the construction of a 2 X 2 table will be excluded from this review.

#### Participants

We will include studies of participants diagnosed with COPD that have been complicated by acute exacerbation of any severity. We will include studies involving participants of any age greater than or including 18 and any gender. In studies where participants are not limited to adults with COPD, only data pertaining to this patient groups will be reviewed.

We will exclude studies involving participants with stable COPD.

#### Index tests

Our index test is sputum colour as a marker for the presence of bacteria in the sputum of adults with AECOPD. We will include studies in which sputum colour has been macroscopically assessed by a professional health care worker or reported by patients. Professional health care workers will for this purpose include doctors, nurses, physiotherapists, respiratory therapists and laboratory based medical scientists. An index test will be considered positive if sputum colour is assessed as “purulent”, “green”, “yellow” and “brown”. An index test will be considered negative if sputum colour is assessed as “mucoid”, “colourless”, “grey” or “white”.

We will exclude studies in which the sputum is only described microscopically and in which the assessor of sputum colour is not well defined.

#### Target conditions

AECOPD secondary to bacterial infection is the target condition. This is defined as the acute worsening of respiratory symptoms, in a patient with COPD, which requires further therapy and is likely due to bacterial aetiology [5].

#### Reference standards

The reference standard is the detection of potentially pathogenic bacteria on bacterial culture of an adequate sputum sample. A sputum sample will be regarded as adequate if it satisfies either the Murray-Washington or Bartlett microscopic assessment criteria. The Murray-Washington criteria define an adequate sputum specimen by the presence of less than 10 squamous epithelial cells per low-power field [21]. The Bartlett criteria derives a score based on the number of neutrophils per low-power field, the presence of mucous strands and the number of squamous epithelial cells per low-power field [22]. A score of 1, 2 or 3 defines and adequate sample [22].

### Search methods for identification of studies

#### Electronic searches

We will search the following databases for studies to be included in this systematic review: MEDLINE, Scopus, Google Scholar Web of Science, Africa-Wide, CINAHL, EMBASE, Health Source Nursing Academy and Cochrane. We will use a combination of Medical Subject Heading (MeSH) terms and free text terms on these platforms. For databases such as CINAHL and EMBASE, which have their own thesauri, controlled vocabulary terms will be translated as appropriate. All databases will be searched from inception until present. A draft search strategy for the MEDLINE search (using PubMed) is provided in Additional file 2.

#### Searching other resources

We will review citation indexes and the references lists of the studies identified through the electronic search for additional articles not found during the initial search. We will also conduct a grey literature search to include conference papers, theses and other unpublished papers (Global Index Medicus, OpenGrey, OpenUCT, OpenDoor, ProQuest dissertations and Theses Global).

The Preferred Reporting Items for Systematic Reviews and Meta-Analyses literature search extension (PRISMA-S) guideline will be used in reporting on the search strategy in the final review [23].

### Data collection and analysis

The screening process and study selection will be completed in accordance with the guidelines published in the *Cochrane Handbook for Systematic Reviews of Diagnostic Test Accuracy* [24].

#### Selection of studies

We will use Rayyan QCRI to assist with the screening of titles and abstracts [25]. Two primary reviewers will independently screen all titles identified by the search strategy. The reviewers will complete a standardised coding sheet (Google Forms) indicating whether a study has met the inclusion criteria or the reason a study has been excluded. Duplicated studies will be removed. The more recent publication with the most complete dataset will be included in the event that duplicate publications for the same data are reported.

The reviewers will select studies from the search strategy in two phases:

#### Phase 1: screening of titles and abstracts

The primary reviewers will evaluate all titles and abstracts from the search strategy against the predetermined inclusion criteria. The full text of a study will be reviewed if it is not apparent from the title and abstract whether a study has met the inclusion criteria, or if both primary reviewers do not exclude the study.

#### Phase 2: screening of full-text studies

The full text of all potentially eligible studies will be reviewed. A third reviewer will adjudicate any discrepancies between the primary reviewers. The reasons for the exclusion of studies will be documented and presented in a table of excluded studies.

### Data extraction and management

Two primary reviewers will develop a data extraction form using Google Forms and will independently extract data from all studies fulfilling the eligibility criteria. The data extraction form will be piloted on at least three potentially eligible studies. A third reviewer will adjudicate any discrepancies between the primary reviewers. The following data will be extracted from the included studies:

- Study characteristics: authors, year of publication, geographical region of study conduct, study design, sample size, clinical setting, index test, reference standard and funding source.
- Participant characteristics: Age, gender, disease and exacerbation severity.
- Outcomes measures: values of diagnostic 2 × 2 table(s) (number of true positive (a), false positive (b), false negative (c), and true negative (d)) that cross‐classified the disease status (as determined by the reference test) and the index test’s outcome for each index test evaluated in the included studies) and number of inconclusive results.

We will contact the relevant authors of primary diagnostic studies in the event of missing data.

### Assessment of methodological quality

Two investigators will independently evaluate the risk of bias for each article reviewed. Any disagreement with be resolved by a third reviewer. Findings will be reported in accordance with the Quality Assessment of Diagnostic Accuracy Studies (QUADAS-2) tool [26].

QUADAS‐2 is the redesigned and improved version of the Quality Assessment of Diagnostic Accuracy Studies list (QUADAS). It comprises four domains: patient selection, index test, reference standard, and flow and timing. Risk of bias is assessed for each domain, and for the first three domains, a statement on concerns regarding applicability is given. Each key domain has a set of signalling questions to help judge the risk of bias and concerns regarding applicability. Signalling questions are answered as ‘yes’, ‘no’, or ‘unclear’. Risk of bias is rated as ‘low risk of bias’, ‘high risk of bias’, or ‘unclear risk of bias’. Concerns regarding applicability are rated as ‘low’, ‘high’ or ‘unclear’. In Appendix we provide the precise criteria, tailored for this review, with which we expect to assess risk of bias and applicability. The Grading of Recommendations Assessment, Development and Evaluation (GRADE) approach will further be used to grade the quality of evidence and the strength of recommendations for sputum colour as a marker for bacteria in AECOPD [27].

### Data synthesis and statistical analysis

Data synthesis and statistical analysis will be completed in accordance with the guidelines published in the *Cochrane Handbook for Systematic Reviews of Diagnostic Test Accuracy* [28].

A descriptive overview of included studies will be presented through two tables. The first table will summarize study design, participant characteristics, details of the index test, details of the reference standard and summary statistics including sensitivity, specificity, true positives, false positives, true negatives and false negatives. The second table will summarize the quality of the included studies, as per the QUADAS-2 framework. We will aim to perform a meta-analysis of the data from included studies, however, the included studies will be synthesized through descriptive analysis only should the data not be amenable to meta-analysis. The Synthesis Without Meta-analysis (SWiM) guidelines will be adhered to in reporting the data synthesis should meta-analysis not be possible [29]. Factors which may preclude meta-analysis may include methodological differences between studies, a general sparsity of data or most studies being assessed as high risk of bias.

Our analysis will be conducted at the level of the sample and not at the level of the participant. For example, if a participant in a study produces more than one sputum sample, each sample will be regarded as an independent index test. We will plot the identified sensitivities and specificities for each index test on Forest plots using RevMan [30]. This will allow for visual examination of variation in test accuracy across studies. We will aim to use values reported in the diagnostic 2 X 2 for each included study. If these data are unavailable, we will attempt to reconstruct these values from reported summary measures. Studies with incomplete or inconclusive index test results will be excluded from statistical analysis but will still be summarized in the descriptive analysis. The results of the index test will be dichotomous; “Positive” or “Negative” based on colour of the sputum. Meta‐analysis using a bivariate logistic regression model with random effects will be conducted around this common threshold (Positive/Negative) using the xtmelogit function in Stata V.14.2 (Stata Corp, College Station, Texas, USA). This will estimate pooled sensitivity and specificity (with 95% confidence intervals).

### Investigations of heterogeneity

We will explore heterogeneity through the visual examination of the forest plots of sensitivities and specificities and through the inclusion of possible sources of heterogeneity as covariates in a meta-regression model if sufficient studies are included in the analysis. We will investigate the following sources of heterogeneity:

- Study setting, that is, outpatient versus inpatient.
- Antibiotic uses, that is, if patients were exposed to antibiotics within 4 weeks of participation in the study; “Yes” if patients were antibiotic exposed and “No” if patients were not antibiotic exposed,
- Source of index test, that is, if sputum colour was assessed by a healthcare professional or patient reported; “Yes” if sputum colour was assessed by a healthcare profession and “No” if sputum colour was patient reported.

### Sensitivity analyses

We will conduct sensitivity analyses to explore the influence of study quality on our findings, drawing primarily on our assessment of study bias using the QUADAS-2 tool. We will first explore the effect of excluding studies in which the index test or reference standard domains are judged as having a high risk of bias or unclear risk of bias as these are considered the most relevant sources of bias. We will then explore the effect of excluding studies in which two or more domains of the QUADAD-2 are judged as having a high risk of bias or unclear risk of bias. We will also use sensitivity analyses to explore the effect of potentially influential studies, such as studies with accuracy estimates markedly different from the rest of the included studies.

### Assessment of reporting bias

We will not undertake any formal assessment of reporting bias in our review due to current uncertainty about how to assess reporting bias in reviews of diagnostic test accuracy, particularly in the presence of significant heterogeneity [28].

## Discussion

This systematic review and meta-analysis will synthesize the evidence on sputum colour as a marker for bacteria in the sputum of adults with AECOPD from the existing literature. The findings of this review may be of interest to clinicians, particularly in resource-limited settings, who may not have access to biochemical, radiological and microbiological special investigations. Furthermore, the findings of this review may have implication for antibiotic stewardship in AECOPD.

### Supplementary Information

**Additional file 1.** PRISMA-P 2015 Checklist.

**Additional file 2.** MEDLINE/PubMed Search.

## Data Availability

Data sharing is not applicable to this article as no datasets were generated or analysed during the current study.

## Appendix

### Review-specific tailoring of QUADAS-2

#### Domain 1: patient selection

***Risk of bias: could the selection of patients have introduced bias?***

**Signalling questions and answer guidelines**

1. Was a consecutive or random sample of patients enrolled?
  We will score ‘yes’ if the study enrolled a consecutive or random sample of eligible participants; ‘no’ if participants were enrolled by selection or convenience; and ‘unclear’ if the study did not report how participants were enrolled.
2. Was a case–control design avoided?
  We will score ‘yes’ if studies are not case–control studies; ‘no’ to studies which are case–control studies and ‘unclear’ if the study design is not reported or we are unable to discern from the text.
3. Did the study avoid inappropriate exclusions?
  We will score ‘yes’ if studies enrolled all patients with AECOPD. We will score ‘no’ if studies excluded patients based on sex, race, presence of fever, disease severity, previous hospitalizations, previous antibiotic use or previous corticosteroid use as these exclusions significantly reduce the generalizability of a study’s findings. We will score ‘unclear’ if studies do not report exclusion criteria or we are unable to identify from the text.

***Applicability: are there concerns that the included patients and setting do not match the review question?***

We will score ‘low concern’ in studies of patients with AECOPD in any setting. We will score ‘high concern’ if studies inappropriately include participants with chronic lung diseases other than COPD (i.e. asthma, bronchiectasis, interstitial lung disease and lung cancer), pneumonia and congestive cardiac failure. We will score ‘unclear concern’ if we are unable to identify if a study’s included patients do not match our review question.

#### Domain 2: index test

***Risk of bias: could the conduct or interpretation of the index test have introduced bias?***

**Signaling questions and answers guidelines**

1. Were the index test results interpreted without knowledge of the results of the reference standard?
  We will score ‘yes’ in studies where sputum colour was reported blinded to the result of bacterial culture on sputum or if it clear that sputum colour was reported before the results of bacterial culture were available. We will score ‘no’ if sputum colour was reported by an individual to whom the results of bacterial culture of the sputum were known. We will score ‘unclear’ if we are unable to identify whether sputum colour was reported with knowledge of the result of bacterial culture from the text.
2. If a threshold was used to define positivity, was it prespecified?
  We will score ‘yes’ in studies which used colour scales to measure sputum colour and which prespecified a colour or a number on the scale which would indicate a positive test. We will score ‘no’ in studies which utilize colour scales to measure sputum colour but do no specify which value on the scale defines a positive test. We will score ‘unclear’ in studies which do not use colour scales to define test positivity.

***Applicability: is there concern that that the index test, its conduct or interpretation differs from the review question?***

We will score “low concern” in studies in which sputum colour is assessed macroscopically from a freshly expectorated sputum sample, by either a health care worker or self-reported by a patient. We will score ‘high concern’ if there is delayed assessment of sputum samples or if sputum colour is reported by anyone besides a treating healthcare worker or the patient. We will score ‘unclear’ if we are unable to determine how the index test was conducted or interpreted.

#### Domain 3: reference standard

***Risk of bias: could the reference standard, its conduct, or its interpretation have introduced bias?***

**Signalling questions and answers guideline**

1. Is the reference standard likely to correctly classify the target condition?
  We will score ‘yes’ in studies where the reference standard is bacterial culture of an appropriately collected and sufficient quality sputum sample according to the Murray-Washington and/or Bartlett criteria. The Murray-Washington criteria define an adequate sputum specimen by the presence of less than 10 squamous epithelial cells per low-power field. The Bartlett criteria derives a score based on the number of neutrophils per low-power field, the presence of mucous strands and the number of squamous epithelial cells per low-power field. A score of 1, 2 or 3 defines and adequate sample. We will score ‘no’ in studies where the reference standard is any medium other than bacterial culture of a sputum sample or if bacterial culture is performed on inadequate sputum samples according to the Murray-Washington and/or Bartlett criteria. We will score ‘unclear’ if the we are unable to identify the nature of the reference standard.
2. Were the reference standard results interpreted without knowledge of the results of the index test?
  We will score ‘yes’ in studies where the results of bacterial culture of a sputum sample were interpreted by someone who was not responsible for defining the colour of the sputum sample and was not aware of whether the sample had been labelled as “positive” or “negative”. We will score ‘no’ in studies where the same individuals who defined sputum colour interpreted the results of bacterial culture on a sputum sample or in studies where the individuals interpreting the results of bacterial culture of a sputum sample were aware if the sample has been labelled as “positive” or “negative”. We will score ‘unclear” if we are unable to tell.

***Applicability: is there concern that the target condition as defined by the reference standard does not match the review question?***

We will score ‘low concern’ in studies where the reference standard is a bacterial culture of an adequate sputum sample, with a prespecified threshold for culture positivity. We will score ‘high concern’ in studies where bacterial culture is performed on inadequate sputum samples. We will score ‘unclear’ if there is not prespecified threshold for culture positivity or in studies where the quality of sputum samples is not described.

#### Domain 4: flow and timing

***Risk of bias: could the patient flow have introduced bias?***

**Signalling questions and answers guideline**

1. Was there an appropriate interval between index test and reference standard?
  We will score ‘yes’ in studies in which sputum samples were processed for bacteria culture on the same day as which the colour of the sample was defined. We will score ‘no’ in studies in which sputum samples were processed for culture on different days than when the colour of the samples was defined. We will score ‘unclear’ if we are unable to identify to interval between index test and reference standard.
2. Did all sputum samples receive the same reference standard?
  We will score ‘yes’ in studies were all adequate sputum samples which were defined as “positive” or “negative” were processed for bacterial culture. We will score ‘no’ in studies were not all adequate sputum samples for processed for culture. We will score ‘unclear’ if we were unable to tell.
3. Were all sputum samples included in the analysis?
  We will score ‘yes’ in studies where the number of adequate sputum samples collected is equal to the number of samples included in the 2 × 2 analysis table, or in which a sufficient explanation is provided for any discrepancy. We will score ‘no’ is studies where the number of adequate sputum samples collected does not equal the number of samples in the 2 × 2 analysis table and no sufficient explanation for the discrepancy is provided. We will score ‘unclear’ if we were unable to tell.

### Judgments for overall ‘risk of bias’ assessments for domains

If we answer:

- all signalling questions for a domain “yes,” then we will judge risk of bias “low”;
- all or most signalling questions for a domain “no,” then we will judge risk of bias “high”;
- one signalling question for a domain “no,” we will discuss with a third author the ‘Risk of bias’ judgement;
- all or most signalling questions for a domain “unclear,” then we will judge risk of bias “unclear”;
- only one signalling question for a domain “unclear,” we will discuss with a third author the ‘Risk of bias’ judgement for the domain.

## Abbreviations

COPD: Chronic obstructive pulmonary disease
AECOPD: Acute exacerbation of chronic obstructive pulmonary disease
