# Supplementary material for: Sputum colour as a marker for bacteria in acute exacerbations of COPD: protocol for a systematic review and meta-analysis
Source: Syst Rev. 2021 Jul 27;10:211. doi: 10.1186/s13643-021-01767-6 (PMC8317370; doi:10.1186/s13643-021-01767-6)
Supplement: Supplementary file 2 — Additional file 2. MEDLINE/PubMed Search. [file 13643_2021_1767_MOESM2_ESM.docx]

| ***Population: adults with chronic obstructive pulmonary disease (COPD) or asthma with an exacerbation*** | | |
| --- | --- | --- |
| *1* | *MeSH terms:* | *Pulmonary Disease, Chronic Obstructive* |
| 2 | *Free text:* | *COPD OR “chronic obstructive pulmonary diseas*” OR “chronic obstructive airway diseas*” OR “chronic airflow obstruction” OR bronchitis OR emphysema OR “chronic respiratory infection” OR “obstructive lung diseas*” OR COAD OR “airway obstruction” OR “bronchus obstruction” OR “lung inflammation”* |
| ***Index test: assessment of sputum colour*** | | |
| 3 | *MeSH terms:* | Sputum |
| 4 | *Free text:* | *Sputum* OR “productive cough”* |
| 5 | *MeSH terms:* | *Color* |
| 6 | *Free text:* | *Colour OR purulent OR purulence OR suppuration OR suppurative OR mucoid OR pigmentation OR green OR brown OR grey OR yellow OR white* |
| 7 | *3 OR 4* |  |
| 8 | *5 OR 6* |  |
| 9 | *7 AND 8* |  |
